# Navigating School Meal Environments: Perspectives of Pupils Diagnosed With Autism Spectrum Disorder or ADHD

**DOI:** 10.1177/10598405251319982

**Published:** 2025-02-24

**Authors:** Susanna G. Sandberg, Mikaela Nyroos, Maria Waling, Cecilia Olsson

**Affiliations:** 1 Department of Food, Nutrition and Culinary Science, 8075 Umeå University, Umea, Sweden; 2 Department of Applied Educational Science, 8075 Umeå University, Umea, Sweden

**Keywords:** school lunch, school restaurant, lunchroom, education, learning environment, neurodevelopmental disorders, disability studies, school nurses, school cafeteria, school nursing

## Abstract

Busy and unstructured school environments can present challenges for pupils diagnosed with autism spectrum disorder (ASD) and attention deficit hyperactivity disorder (ADHD). Although school restaurants may be demanding, limited research has focused on these pupils. This study explores how pupils diagnosed with ASD or ADHD navigate the physical, social, and pedagogical environments of school meals. Based on ethnographic fieldwork in four Swedish schools, involving observations, conversations, and interviews with five 12-year-old boys and their mothers, findings show how pupils valued having a teacher or classmate nearby during lunchtime. Crowded and narrow spaces posed motor challenges, leading to spills and comments on table manners. Socially, pupils alternated between engaging with others and seeking solitude to escape noise and interactions. The study calls for reflection on how societal norms and environmental structures of school meals impact pupils diagnosed with ASD or ADHD, emphasizing the role of school nurses in identifying potential issues.

## Introduction

Neurodevelopmental disorders, such as autism spectrum disorder (ASD) and attention deficit hyperactivity disorder (ADHD), have gained increased public, political, and academic attention worldwide in recent decades (Cainelli & Bisiacchi, 2022). In particular, in the context of inclusive education, there has been an increasing concern and debate on, the role of the school and its constituent environments in relation to neurodevelopmental disorders (Cainelli & Bisiacchi, 2022; Erlandsson & Punzi, 2017; Ponnou & Gonon, 2017). For example, a Swedish study found that pupils diagnosed with ASD and ADHD often dislike school environments and feel unheard compared to peers (Beckman et al., 2016).

Despite growing interest in neurodevelopmental disorders and school environments, the school meal setting has largely been overlooked (Bashinski & Smilie, 2018). Despite growing interest in neurodevelopmental disorders and school environments, the school meal setting has largely been overlooked (Bashinski & Smilie, 2018). In Sweden, as well as in other European countries, the term school restaurant is commonly used (Berggren et al., 2019; Lalli, 2020). Swedish National Guidelines for School Meals (SFA, 2023) employ this term, with the intention of elevating the status of this learning environment within schools (Persson Osowski & Fjellström, 2018). School restaurants have been reported as stressful, crowded, and noisy by pupils diagnosed with ASD or ADHD (Hebron & Humphrey, 2012; Hill, 2014). These pupils can also struggle in sensory-challenging learning environments such as those with high noise levels and bright lights (Tufvesson & Tufvesson, 2008). Additionally, feeding difficulties are commonly associated with ASD and ADHD (Smith et al., 2020), and socializing during meals can be stressful for those diagnosed with ASD (Saggers et al., 2012).

With school meal programs now common worldwide (Global Child Nutrition Foundation, 2022), it is crucial to explore how the school meal environment impacts children diagnosed with ASD or ADHD. The aim of this research was to study how pupils diagnosed with ASD or ADHD, experience and navigate the physical, social, and pedagogical environments of the school meal.

In Sweden, the context of the present study, tax-funded, nutritious meals are provided to all pupils in compulsory school (Gullberg, 2006), and are regulated by the Swedish Education Act (2010). These meals, eaten in designated school restaurants, are considered to be a part of the educational process (Government's Bill 2009/10:165) and are referred to as “pedagogical meals” in the National Guidelines for School Meals (SFA, 2023). As part of this, it is suggested that teachers eat with pupils, act as role models, and teach them about healthy eating. Previous research indicates that engaging other school personnel, including school nurses and meal staff, can be beneficial, as lunchtime provides opportunities for informal conversations in nonclassroom-settings (Frödén & Pettersson, 2021; Lintukangas & Palojoki, 2015).

The National Guidelines for School Meals further emphasize nutritional value, a pleasant environment, as well as social and environmental sustainability (SFA, 2023). School meal environments are seen as places for positive social interactions (Askelson et al., 2022; Berggren et al., 2019; Bruselius-Jensen, 2014) but can also be noisy, stressful, and potentially risky with exposure to assault and bullying (Berggren et al., 2019; Horton & Forsberg, 2020; Tørslev et al., 2017); causing distress related to overcrowding (Moore et al., 2010), and including feelings of insecurity (Borg, 2023).

The present study focuses on pupils diagnosed with ASD or ADHD, both developmental disorders that overlap in terms of difficulties related to communication, social interactions, planning and organizing activities (Antshel & Russo, 2019), as well as motor skills (Kaiser et al., 2015; Kaur et al., 2018). These challenges can make social activities or navigating areas where many people are gathered, such as schools, problematic. Previous research has shown that unstructured and crowded school environments can cause anxiety and stress for these pupils, with school restaurants being particularly problematic (Hebron & Humphrey, 2012; Humphrey & Lewis, 2008; Penney, 2013; Saggers et al., 2012). Loud noises can also lead to concentration problems (Saggers et al., 2012; Tufvesson & Tufvesson, 2008). Pupils diagnosed with ASD have been reported bypassing the queue or bringing packed lunches to avoid this busy area (Hill, 2014) or having lunch at off-peak times or in separate rooms (Frödén & Pettersson, 2021). Pupils diagnosed with ADHD or ASD also have described themselves as “different” from their peers, facing socialization challenges or potential bullying at school (Gibbs et al., 2016; Penney, 2013; Wiener & Daniels, 2016), and may therefore choose solitude during lunchtime (Saggers et al., 2012).

According to the Swedish Education Act (SFS, 2010: 800), all pupils have the right to receive support to fulfill their educational goals based on their individual needs. The legislation provides additional support with pupil health teams, positioned at the intersection of learning and health, and providing medical, psychological, and psychosocial, and special needs support. The team is tasked with promoting pupils’ health and learning and contributing to supportive learning environments. Within this team, the school nurse serves as a medical resource and conducts health checks, dialogs, vaccinations, and health education (National Board of Health and Welfare, 2024). With regard to support for pupils diagnosed with ASD or ADHD, the school nurse is seen as an important link, cooperating with other school staff and caregivers (Golsäter et al., 2017; Salminen et al., 2024), to detect pupils’ stress at an early stage and work as an additional safe adult (Berglund Melendez et al., 2020).

### Theoretical Framework

This study embraces a social constructionist perspective as outlined by Burr (2015), viewing disability as influenced by social criteria rather than solely biology. The classification of bodily “functions” is relational and context-dependent, often based on able-bodied norms (Andrews et al., 2000; Bylund, 2020). In disability studies, two main conceptualizations exist: the dominant psychomedical model, which focuses on individual deficiencies and to compensate for these shortcomings (Baglieri, 2023), and the social model (Baglieri, 2023; Nilholm, 2020), which sees disability as a product of social and cultural construction, dependent on environmental factors rather than individual deficiencies. This study aligns with the social model, emphasizing that environmental design and societal norms contribute to challenges faced by individuals labeled with a disability while not denying the existence of individual impairments (Andrews et al., 2000; Baglieri, 2023). For instance, pupils diagnosed with ASD or ADHD may struggle during school meals not because of their disability but due to distracting noises or unclear instructions (Bylund, 2020).

This study uses three interconnected aspects of the school meal environment: the physical, social, and pedagogical space—as a theoretical lens, aligning with previous research (Lai et al., 2020; Leifler et al., 2021; Rönnlund et al., 2020). The physical space includes the arrangement of the room, for example, furnishings, light, and sounds (Beckers et al., 2016; Closs et al., 2022). The social space concerns pupil safety and cooperation (Westling Allodi, 2010), and the pedagogical space concerns, for instance, teacher support and resources for learning (Closs et al., 2022).

## Methodology

### Study Design

This article is based on a 5-month period of participant observations, informal conversations, and semistructured individual interviews with pupils diagnosed with ADHD or ASD and, separately, with their mothers. Using an ethnographic methodology approach, fieldwork was carried out in four Swedish compulsory schools with the aim of understanding participants’ everyday lives in their natural environment over an extended time period (Hammersley & Atkinson, 2019). Ethnography traditionally focuses on a few cases or settings using a variety of methods to deeply understand a phenomenon's complexity (Hammersley & Atkinson, 2019).

### Getting Access to the Field and Recruitment

Pupil recruitment began in the autumn of 2021 when school nurses in a Swedish municipality were contacted and briefed about the study. Chosen as gatekeepers due to their role in managing adjustments during school meals for pupils diagnosed with ASD or ADHD, all nurses expressed interest in assisting recruitment. To gain access to schools, 17 head teachers were informed, and nine agreed to participate. The briefed school nurses identified eligible pupils aged 11 to 13 years (in grades 4–6) and diagnosed with ASD and/or ADHD (with absence of intellectual disability) as inclusion criteria. The school nurses contacted nine caregivers and provided study information for both caregivers and children. At this stage, all personal information remained anonymous to the researchers. Five pupils from four schools agreed to participate, and their caregivers provided written consent.

Each pupil's class teacher was then contacted and provided with information about the study, requesting permission to observe classes and join the pupils for lunch. During the first classroom visit, all pupils were told that the researcher (first author/SS) would be present for a few weeks, and their parents were informed using the teacher's weekly newsletter.

### Observing and Documenting the Setting

The observations were conducted by the first author [SS], who was trained through pilot observations prior to the start of this study. Fieldwork took place between February and October 2022, involving each participating pupil for about one calendar month (16–20 school days), 1 to 3 h per visit. Observations typically began in the classroom before lunch, then accompanying the participant, their class, and teachers to the school restaurant, where the researcher ate lunch with them. On some occasions, the researcher was also present during lunch breaks and afternoon classes.

The observation technique initially involved “casting the net” broadly to gain general impressions of the school restaurant, its people, and events (Emerson et al., 2011). This approach included further attention to the physical environment (e.g., decorations, sounds, and furnishings), the social environment (e.g., conversations and invitations) and the pedagogical environment (e.g., information and support). Throughout the fieldwork, the researcher engaged in informal conversations with the participants, which were also documented.

Fieldnotes were initially captured in memory (“head notes”), and as brief notes on paper or a mobile phone (“jottings”), following Emerson et al. (2011). Directly after each observation, the jottings were reconstructed into comprehensive narratives, with daily observation resulting in approximately 700 to 2000 words of text. For respondent anonymity, all participants were given pseudonyms, used consistently both in fieldnotes and this article.

### Interviewing Pupils and Mothers

Following Cederborg (2009) and Magnusson and Marecek (2015), a semistructured interview guide was used when interviewing pupils individually. The guide included open-ended questions (e.g., “Tell me about X”) and closed questions (e.g., “Do you like X?”). This combination was based on previous research indicating that children diagnosed with ASD often find abstract questions challenging to answer (Fayette & Bond, 2017; Tyrrell & Woods, 2018).

Four interviews were conducted with pupils in a separate room near the classroom during the regular school day. One interview took place at the pupil's residence, with the pupil's mother present for support, as per the pupil's preference. To maintain the pupil's engagement and alertness, the interviews were divided into two sessions (Kelly, 2007). The total interview time for each pupil was between 30 and 50 min. Photos taken by the researcher, capturing the school restaurant entrances, halls, serving areas, seating, and dish delivery zones, were used during interviews to facilitate discussions and serve as visual tools to help the pupils explain and develop their thoughts about the environment. Paper and colored pens for drawings were also provided at all interviews (Tinson, 2009). However, none of the participants chose to use them.

Participants’ mothers were interviewed after the fieldwork at each school was completed. Four interviews were conducted at the university, and one was conducted digitally. Using a guide, the interviews lasted between 45 and 80 min. All interviews with participants and their mothers were digitally recorded and transcribed verbatim.

### Analytical Procedure

Data analysis followed the thematic analysis principles outlined by Braun and Clarke (2022). Initially, all interviews were listened to, and transcripts were read for familiarization. Interviews were then initially coded inductively with data analysis software MAXQDA (version 22.8.0), where segments of text or sentences from the transcripts were coded at the semantic (descriptive) or latent (interpretive) level. Observational fieldnotes were also read for familiarization, manually coded, and the codes transferred into charts in a Microsoft Word document. Codes from interviews and observational field notes were then sorted into clusters, where codes representing similar phenomena were grouped together and assigned a distinct label at a more abstract level. For example, the codes “Support to be on time” and “Help to serve oneself food” with others were grouped within the cluster “The need for support”.

Connections and associations among clusters within the dataset were then identified and organized into a mind map, and candidate themes were formulated. All authors—with diverse expertise in food, nutrition, child and adolescent psychiatry, and education, including experience in qualitative child research—were familiar with the empirical material. The code structure, clusters, and candidate themes were discussed and agreed upon within the research group. Discussion contributed to modification, refinement, and reorganization into three themes.

### Ethical Considerations

The study received approval from the Swedish Ethical Review Authority (ref. no. 2021-05081). Informed consent was obtained at three levels (head teacher, caregiver, and pupil). Due to the study's focus and methodology, additional ethical precautions were taken. Given that the participating pupils had been diagnosed with a neurodevelopmental disorder, personal data were considered sensitive (GDPR 2016/679). Therefore, to protect the integrity and confidentiality of the data, the pupil's diagnosis was never discussed in interviews or during fieldwork unless raised by the pupil. Classmates were informed only that the study concerned school meals, with no further details provided.

To minimize potential stress during interviews, they were scheduled for the last week of fieldwork at each school, ensuring participants were familiar with the researcher (Fayette & Bond, 2017; Tinson, 2009). Throughout, consent was consistently sought from the pupils, whether for the researcher's presence during meals or for the interviews (Tinson, 2009). Additionally, regular telephone contact was maintained with caregivers to address any concerns about the pupils’ potential discomfort.

## Findings

### Participating Pupils and the Setting

Of the 12-year-old boys participating, given the pseudonyms Alve, Morris, Tim, Olof, and Simon, two were diagnosed with ASD, and three were diagnosed with ADHD. They attended schools in a small municipality in Sweden, with pupil populations ranging from less than 100 to 800. One school was in the countryside, while the others were near the largest urban area.

The school restaurants had seating capacities of 50 to 200 people and were frequented by pupils, teachers, and occasionally other school staff like the head teacher or the school nurse. Some participating pupils had a fixed lunchtime daily, while others had varying times based on a weekly schedule. Three schools had fixed seating with reserved tables but not designated chairs, while one school had open seating.

All schools provided a daily main hot dish, crisp bread, spreads, and a salad buffet with at least five vegetable options. Pupils served themselves from a long trolley with utensils, plates, and napkins. Beverages and glasses were available at a nearby station.

## Identified Themes

### Ensuring Closeness to a Guiding Hand

The theme *Ensuring closeness to a guiding hand* captures how the boys valued maintaining a sense of security and support in the school lunch context. As such, their strategies involved staying close to school staff or classmates to ensure they were not left behind and to keep themselves on the right track. Staying close to a familiar person was also interpreted as being valuable for establishing routine and predictability.

An example of ensuring closeness to a safe person was seen when some participating boys stayed behind after class to walk with their teacher to lunch. While classmates rushed ahead to be first in line outside the restaurant door, participants like Alve lingered, seemingly preferring the teacher's company and regularly choosing to sit with them in the school restaurant. In his interview, Alve mentioned his preference for sitting with teachers:

*SS: Do teachers usually sit with you at the table?*

*Alve: Yes, we usually take a seat where there are teachers.*

*SS: Mmm. Why do you do that?*

*Alve: It's fun.*

When Alve sat at the table and wanted to refill his plate (as he often did), it seemed that he wanted to ensure that a teacher or his classmates would still be at the table when he returned so he would not be left by himself. The following observational fieldnote discloses this intention:

*Alve looks around, glancing at the plates of those sitting at the table. Some of those seated around the table got up to leave. […] Alve then half-rises to get more food but pauses in his step and says to me [SS]: “you'll have to wait for me” and I [SS] respond: “of course”.*

Another example of how the participating boys sought support was seen in the case of Olof. As with Alve, he often chose tables where teachers were seated, preferably those with whom he had a closer connection. Olof explains this in an interview:

*SS: Is there usually anyone else sitting around the table?*

*Olof: No one else… or I sit with… with some teachers.*

*SS: Do you usually sit where there are teachers?*

*Olof: Only teachers, but I… well… yes…*

*SS: Yeah, there are teachers also at the table?*

*Olof: Yeah… always those that I … the ones I know. Not strangers, I don't know.*

*SS: Not strangers, right? You usually sit with teachers that you know?*

*Olof: Yeah, always, yes.*

Besides sitting with teachers, Olof accompanied a classmate, who apparently played a significant role for Olof during lunch, such as providing that extra “push” to help him make decisions. For example, the classmate encouraged Olof to refill his plate, get an extra piece of crispbread, or decide when to leave the table. The fact that the classmate often acted as an assistant for Olof was both noted and encouraged by teachers. For example, teachers would say, “*It´s great that [pupil's name] keeps an eye on him*.” The following text segment is from an observation when the classmate was absent for the day, and Olof instead turned to the researcher for support:

*Olof has finished all the pieces of sausage, and there is sauce and lentils left on the plate. I [SS] ask if he is full, and he answers no. He then says that he should have taken fish instead. I [SS] suggest that he could go and get fish now. He sits quietly for a moment and then asks, “Should I get a new plate then?” I [SS] say, “Yes, that might be best,” and “Take the old plate to the counter”. He stands up, hesitates for a moment, and then asks, “Should I use the same utensils?” I [SS] nod and tell him that it's fine. After a short while, he returns without a plate. I [SS] ask if the fish had run out. He nods, stands by the table for a moment, but then takes his utensils and goes to return them to the counter.*

Most of the mothers talked about their children having difficulties visualizing what to do next and emphasized the importance of having someone offer direction. Some of the participating boys had been assigned teacher assistants in lower grades who supported them during lessons, breaks, and meals. While this formal support was no longer deemed necessary at their current age, the need for informal support and someone to offer an extra “push” was emphasized by Tim's mother, who explained it as follows:

*That they [the teachers] could perhaps sit there… at the table where he sits and nudge him, like “hey, you need to eat a bit more now, you can't just sit and look at that, or chat with others”. Because they eat while talking to each other… but he's just so… “when I talk, I don't eat”, and suddenly he's alone and everyone else is gone because they've finished eating and he's left with his food. So, it would certainly have been good if someone had sat there and nudged him a bit more.*

A few of the mothers developed strategies to ensure their child received guidance regarding the school meal. For example, they set alarms on their sons’ cell phones to remind them to eat lunch on time, or prepared a food box the child could take to school in case they missed the meal.

### Managing the Body in a Challenging Environment

The theme *Managing the body in a challenging environment* describes how struggles with motor skills affected the participants, both in navigating the crowded school restaurant and while eating. Furthermore, actions like moving around and talking to peers often distracted them from finishing lunch.

Many school restaurants were cramped for space, with many tables and chairs arranged in rows close together. In Alve's school, most tables were joined together to accommodate as many diners as possible. These rows required everyone to squeeze between them to reach a seat in the middle of the row, which often resulted in bumping the backrests of occupied chairs. For Alve, navigating through this crowded space, and carrying everything to the table often seemed to be an awkward balancing act. Carrying often led to beverage or food spills on the floor, or on the table at arrival. Difficulties were also observed with the use of utensils. When Alve faced a dish difficult to scoop with a fork, he sometimes poured sauce directly from the plate into his mouth, which led to spills. Field notes reveal that teachers often commented on these spills, viewing them as bad manners. Some boys were aware of this and joked about it like Morris did during one lunch:

*“Don’t sit with me when I eat bulgur” and laughs. I [SS] ask why, and he makes a sweeping gesture with his arm, demonstrating himself eating. I [SS] ask if food ends up everywhere, and he laughs and says, “yes”*.

All mothers mentioned that their children had motor skill differences compared to peers, affecting meals since they were young. These difficulties with fine and gross motor skills led to challenges like scooping food with a fork, being “clumsy” while eating, and having trouble serving themselves. Morris’ mother remarked on these eating manners: “*Well, he probably spills more than the average*.”

Apart from the need to balance the plate and other food items *en route* to the table, a high incidence of movement was observed during the school lunches. Frequent movements were evident for all pupils and adults, but it was particularly noticeable for the study participants. The latter regularly walked back and forth many times to the self-service trolley. Typically, during the first round, participants brought a plate with the main dish and utensils to the table. Subsequently, they often took an additional walk for beverages and, perhaps, another one for bread or vegetables. Serving trays to carry all the components were available for the youngest pupils. However, for the middle school pupils, serving trays were not offered, which, according to the school meal staff, was due to the extra load of dishes that it entailed. A few participants mentioned that they sometimes skipped a beverage during lunch to avoid extra trips. Morris’ frequent movements during lunch were noted:

*After a few minutes of sitting, Morris gets up again to refill his bowl with soup. He returns with the bowl, sits down, eats a couple of spoonfuls, then gets up again. He goes to get a glass of water and returns to the table. At the table, ice hockey is being discussed, and which team is better, the Red team or the Green team. (…) Morris then gets up again, refills his bowl with soup, and returns to place the bowl on the table. Still standing, he then goes to get five or six apple slices on a napkin and returns to the table once more.*

Other aspects of movement involved off-task activities during meals, like chatting, joking, and playing, which impacted the participants’ ability to finish their meals at the same time as others. Tim, for example, started eating late due to these activities and often left the table without completing his meal when his peers finished.

### Negotiating Proximity to Social Interactions

The theme *Negotiating proximity to social interactions* illustrates how the pupils navigated social stimuli and ambient noise during meals, sometimes engaging in conversations and sometimes retreating. Eating school lunch often involves sitting with others but the participants occasionally isolated themselves for respite.

Queuing outside the school restaurant was often crowded and noisy, with pupils gathered in disorganized clusters. Narrow entrances at some schools led to inevitable congestion, high social stimuli, and noise levels, making it a potential breeding ground for conflicts. Some participating pupils mentioned disliking or even hating the queuing because of the crowding.

Fixed seating arrangements in most school restaurants were observed to impact pupils’ social interactions in various ways. For instance, at Morris’ school, pupils were assigned specific tables for their class. Observations showed that Morris often chose an empty table to dine alone, which he elaborated on during an interview:

*SS: Is there usually someone sitting next to you when you eat?*

*Morris: Sometimes…*

*SS: Mmm.*

*Morris: I actually like eating alone.*

*SS: Mmm. So sometimes you sit alone and eat?*

*Morris: Mmm.*

*SS: What do you like about eating alone?*

*Morris: You're alone. It's nice.*

*SS: Mmm… I ask some tricky questions, but what's nice about being alone?*

*Morris: I don't know.*

*SS: No.*

*Morris: It's because… because you're alone. (…) It's quiet.*

Fieldnotes revealed that Morris occasionally chose to sit with his classmates, often after being encouraged by teachers to join others. However, when he did sit with them, he often selected a seat slightly apart from the group, or at the edge of the table. For Morris, and some of the other boys in the study, engaging in conversations carried the risk of social exclusion. In Morris' case, while he sometimes attempted to converse with other pupils, his efforts were often met with minimal responses or eye-rolling from peers. Tim's mother described similar instances of peer victimization during meals:

*But now he has started to notice it himself, like “why do they always do that, like roll their eyes when I say something, or just walk away when I'm trying to say something”. I'm just like, “those aren't good friends, because you listen to them, right?”*

At Simon's school, he and his classmates had fixed seating arrangements in the restaurant. At his table, designed for six people, conversations among his classmates were often lively, but Simon usually sat quietly, appearing to “zone out” from conversations. Occasionally, Simon's classmates tried to include him in their discussions and show care during lunches. In contrast, after the meal when the class dispersed, Simon was often observed wandering alone. A fieldnote from a school lunch with Simon captures this scenario:

*The guy seated in the corner of the table asks Simon where he was yesterday. Simon doesn't respond. The guy asks the others around the table, “Can he hear me?” The girl next to him taps Simon's shoulder and says, “Earth to Simon”, drawing Simon's attention to the guy in the corner asking something. “Where were you yesterday?” he asks again. Simon takes a moment to respond and then says, “The dentist”.*

Most mothers recognized the need to limit social interactions and sensory stimuli to help their children focus on eating, with some suggesting that their child would have benefited from eating in a quieter room. Alve's mother described how he previously required a quiet space with fewer people to cope during meals and how this strategy helped him manage the overwhelming stimuli at school lunch. She noted, “*I think he found it comfortable*.”

Mothers noted the challenge of finding “private” dining spaces, as most schools lacked separate dining rooms. During fieldwork, only one school had such a room, but it was primarily used for storage and as a staff dressing room. Pupils seeking solitude were instead directed to classrooms or small study rooms.

## Discussion

This study aimed to contribute to the knowledge of how pupils diagnosed with ASD or ADHD experience and navigate the physical, social, and pedagogical school meal environment. Findings show that participating pupils valued having a teacher or classmate nearby for support during lunchtime. The school restaurant was generally crowded and lacked space, causing pupils challenges regarding motor skills difficulties, resulting in spills and mess and frequent journeys to gather all food items. Socially, pupils fluctuated between interacting with others and seeking solitude for respite.

Staying close to a teacher or classmate can be seen as a protective strategy in unpredictable environments, which might produce anxiety and stress (Öster et al., 2020). This behavior also reflected uncertainty in navigating lunchtime routines, a feature that may be linked to difficulties in organizing and planning, commonly associated with neurodevelopmental disorders (Antshel & Russo, 2019). The presence of adults in school restaurants has been shown to enhance pupil safety, given that these spaces have been reported as insecure (Borg, 2023; Frödén & Pettersson, 2021; Horton & Forsberg, 2020). Supporting Perfitt (2013), our study highlights the importance for pupils diagnosed with ASD or ADHD of not only having adults present but also having a familiar person nearby in the school environment. Additionally, we found that some participants would have benefited from a designated assistant to provide support during lunch and throughout the school day. Teacher support during meals can also encourage pupils to try new foods (Thorsteinsdottir et al., 2021), addressing the food selectivity commonly identified in children diagnosed with neurodevelopmental disorders (Smith et al., 2020).

Our study found that struggles with coordination and motor skills were common challenges, causing increased spills, multiple trips to gather food because they were unable to carry all items at once, and comments about their upbringing from teachers. These pupils also perceived themselves as having poor table manners. Prior studies have indicated how difficulties with motor skills can cause challenges with utensils for children diagnosed with ASD or ADHD (List Hilton, 2014), and being a more common problem, often draws negative comparison to peers without disabilities (Kaiser et al., 2015; Kaur et al., 2018). Pupils have reported fear of embarrassment and anxiety about unwanted attention when spilling food or stumbling (Berggren et al., 2019; Tørslev et al., 2017). Our findings suggest that these motor skill challenges may increase the likelihood of attracting unwanted attention in school restaurants. Additionally, children with motor skills challenges are at an increased risk for peer victimization (Oksendal et al., 2022).

In our study, observed motor skill struggles can be seen as relational and context-dependent (Baglieri, 2023). Factors like narrow spaces and closely arranged tables, and the absence of serving trays in the school restaurants may have contributed to these challenges. In a school restaurant with more space (or fewer people), trays for carrying food items and spoons to scoop up bulgur or sauce might make motor skills issues less problematic. The lack of trays also led to food spills on tables instead of being contained on the tray. In all the schools in this study, serving trays were provided to younger pupils, while older pupils were expected to manage without serving trays, overlooking differences in individual development or needs. This approach likely aimed at organizational efficiency, as providing trays for all would require more dishes.

The observed high participant rate of off-task activities during school meals, such as moving around and talking to peers, may be linked to ADHD-related hyperactivity and attention difficulties (Antshel & Russo, 2019). Frequent table departures and delayed eating due to talking have been previously observed in ADHD-diagnosed children (Sha'ari et al., 2017).

Regarding the social environment in our study, interactions fluctuated, with some participants choosing solitude or “zoning out” while in the company of others. This behavior, interpreted as a way to block out external stimuli, was seen as a strategy to avoid peer victimization, challenging social situations, and sensory overload, which is consistent with Gibbs et al. (2016), Penney (2013), and Tufvesson and Tufvesson (2008). Some participants had previously been seated in separate rooms for these reasons, reflecting findings by Frödén and Pettersson (2021). However, in contrast, when queuing—which all participants described as something they disliked—their ability to choose solitude was likely limited by the need to remain in the cramped line.

Eating together involves social and cultural expectations, such as socializing, conversing, and adhering to norms (Jönsson et al., 2021). Preferring to eat alone and thus limiting social interaction, may challenge the conventional understanding of meals as an important social practice (Jönsson et al., 2021). Conversely, being required to sit with others can increase children's vulnerability. In our study, Morris had to sit with others at reserved tables, risking peer victimization. The potential of sharing a meal as a vehicle for community-building and belonging was instead perceived as something that demonstrated differences in social position (Fieldhouse, 1995). By contrast, Simon benefited from fixed seating, which encouraged socialization. Simon could participate according to his preferences during meals and was also invited into discussions with peers, which facilitated inclusion. This mirrors findings by Ng et al. (2013), where mixing pupils from different social groups in school during school lunch promoted community and reduced prejudice. In light of this discussion, fixed seating clearly presents a dilemma, as pupils prefer choosing their own seats with peers, they feel safe with (Berggren et al., 2019; Daniel & Gustafsson, 2010).

### Methodological Considerations

In this study, only boys participated. Of nine potential participants contacted by the school nurse, two were girls. The overrepresentation of boys may be due to boys meeting the diagnostic criteria for ASD or ADHD at an earlier age (Klefsjö et al., 2021; Loomes et al., 2017), or that girls show a stronger tendency to mask their interactional deficiencies (Cook et al., 2017). No fathers participated, despite invitations to both caregivers. This aligns with statistics showing that mothers are typically the primary caregivers and the dominant recipients of care allowances for children diagnosed with disabilities in Sweden (Swedish Social Insurance Agency, 2024).

To elicit a child's perspective, participants were encouraged to photograph their school lunch and the school restaurant (Nilsson et al., 2015). However, all boys declined. This might be due to anticipated attention from classmates, difficulties handling a mobile camera, or challenges in understanding the abstract idea of photographing their meal and environment (Cederborg, 2009).

With the small number of participants and specific settings in this study, caution should be taken when considering the transferability of the findings. The study focuses on five boys’ individual experiences and does not aim to represent all children diagnosed with ADHD or ASD. Nevertheless, the study provides useful insights, initiates discussion, and is a foundation for future research.

### Implications for School Nursing

School nurses can play an important role by bringing complementary expertise to the intersection of health services and education, collaborating with head teachers, teachers, and other school staff to address school-related issues (Helleve et al., 2022). In terms of this study, school nurses can help address difficulties faced by pupils diagnosed with ASD or ADHD during school meals. At the individual level, school nurses conduct regular health check-ups, including measurements of weight and height, as well as engaging in health dialogs with pupils. In this role, school nurses can be among the first to identify individual issues related to school meals, as stress and anxiety may have a negative impact on eating (Thorsteinsdottir et al., 2023), potentially leading to somatic symptoms and stunted growth (Inzaghi et al., 2022). Further, school nurses might uncover underlying causes of pain, stress, or anxiety among pupils diagnosed with ASD or ADHD. For example, identifying headaches as a possible result of skipping meals (Berglund Melendez et al., 2020). Thus, school nurse insights can enhance understanding and support strategies to promote wellbeing, engaging in dialog with both pupils and caregivers. At the group level, working alongside other professionals in the pupil health team, school nurses can increase staff knowledge of potential barriers related to the physical, social, and pedagogical aspects of the school meal environment. Such efforts would contribute to the wellbeing of pupils diagnosed with ASD or ADHD and might also foster inclusiveness for all pupils.

## Conclusion

Based on our findings and our theoretical starting point that environmental arrangements and societal norms contribute to challenges faced by individuals labeled with disabilities, we advocate that a safe learning environment during school meals requires the presence of a trusted adult. Additionally, it is important to recognize how narrow spaces, crowding, and the complexity of seating arrangement may impact pupils diagnosed with ASD or ADHD and potentially exacerbate their challenges. Finally, diversity can be embraced through reflection on traditional norms about what constitutes appropriate behavior and “proper” table manners during meals. Our findings suggest the relevance of including pupils’ experiences of the school meal environment in school nurses’ health dialogs since such experiences may impact pupil wellbeing.
